# Group‐based Acceptance and Commitment Therapy (AHEAD) for adolescents with multiple functional somatic syndromes: A randomised trial

**DOI:** 10.1002/jcv2.12047

**Published:** 2021-12-08

**Authors:** Karen Hansen Kallesøe, Andreas Schröder, Jens Søndergaard Jensen, Rikard K. Wicksell, Charlotte Ulrikka Rask

**Affiliations:** ^1^ The Research Clinic for Functional Disorders and Psychosomatics Aarhus University Hospital Aarhus C Denmark; ^2^ Department of Clinical Medicine Aarhus University Aarhus N Denmark; ^3^ Department of Clinical Neuroscience Karolinska Institutet Stockholm Sweden; ^4^ Department of Child and Adolescent Psychiatry Aarhus University Hospital Aarhus N Denmark

**Keywords:** Acceptance and Commitment Therapy, adolescents, functional somatic syndromes, group‐based therapy, randomised controlled trial

## Abstract

**Background:**

Evidence for treatment of adolescents with multiple functional somatic syndromes (FSS) is sparse. This study examined the efficacy of ‘Acceptance and Commitment Therapy for Health in Adolescents’ (AHEAD), a generic group‐based treatment for adolescents with co‐occurrence of multiple FSS.

**Methods:**

A randomized trial was conducted at a specialized university hospital clinic. Adolescents (15–19 years) with multiple FSS of at least 1 year's duration were randomly assigned to AHEAD or enhanced usual care (EUC). AHEAD consisted of nine modules (i.e., 27 h) and one follow‐up meeting. Primary outcome was physical health (SF‐36). Various secondary outcomes and treatment targets were included (e.g., symptom severity, symptom impact, and illness perception). A linear mixed‐effects model was used for analysis. Trial‐registration: ClinicalTrials.gov NCT02346071.

**Results:**

Ninety‐one patients were included. At 12 months, no significant difference in physical health was identified between groups (mean adjusted difference 1.2 [95% CI −1.6 to 4.0], *p* = .404). However, different developments over time were seen with an interaction effect between intervention arm and time (χ^2^(5) = 14.1, *p* = .0148). AHEAD patients (*n* = 44) reported a clinically relevant improvement at end of treatment and at 8 and 12 months, while EUC patients (*n* = 47) displayed a clinically relevant improvement at 12 months. Furthermore, AHEAD patients showed a faster improvement on symptom severity, symptom impact and illness perception. EUC patients received more psychological treatment outside the trial (*p* ≤ .001) than AHEAD patients. Treatment satisfaction with AHEAD was high in contrast to EUC.

**Conclusions:**

Compared with EUC, AHEAD had no additional advantage on the improvement of physical health at the primary endpoint of 12 months. However, a faster improvement of physical health was seen in AHEAD and considerably more psychological treatment was received outside the trial in EUC with clinically meaningful improvements in both groups. The results underpin the importance of an organised and systematic treatment offer for the most severely affected youth.

## Graphical abstract

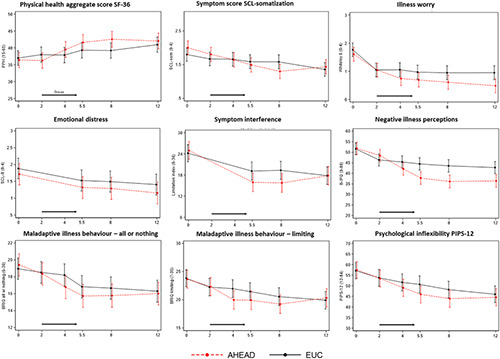

This randomized controlled trial examined the efficacy of ‘Acceptance and Commitment Therapy for Health in Adolescents’ (AHEAD), a generic group‐based treatment for adolescents with co‐occurrence of multiple FSS. Ninety‐one adolescents (15‐19 years) with multiple FSS of at least 1 year’s duration were randomly assigned to AHEAD or enhanced usual care (EUC). Compared with EUC, AHEAD had no additional advantage on the improvement of physical health at the primary endpoint of 12 months. However, a faster improvement of physical health and a range of secondary outcomes was seen in AHEAD.

**Key points**

- Previous interventions for youth with functional somatic syndromes (FSS) have focused on single‐syndrome presentation potentially overlooking the most severely affected youth with co‐occurrence of multiple FSS.
- This is the first randomised trial examining the efficacy of a group‐based psychological intervention for youth with multiple FSS, that is, Acceptance and Commitment Therapy for Health in Adolescents (AHEAD) in comparison with enhanced usual care (EUC).
- AHEAD was not superior to EUC on primary outcome (SF‐36 physical health) at 12 months but showed a faster improvement of physical health, symptom severity, and symptom interference and a larger reduction of negative illness perceptions.
- Patients receiving AHEAD reported a notably higher treatment satisfaction, suggesting feasibility of a generic treatment approach for multiple FSS.
- Additional gains of AHEAD was less use of pharmacological treatment and other non‐documented treatment forms.

**Abbreviations**
ACT: Acceptance and Commitment Therapy
AHEAD: Acceptance and Commitment Therapy for Health in Adolescents
SF‐36: Short Form 36

## INTRODUCTION

Functional somatic symptoms such as fatigue, nausea, dizziness, and pain are prevalent in adolescents with various levels of impairment (Garralda & Rask, 2015). When severe, such symptoms may cause school absence, social withdrawal, and low quality of life with a considerable risk of chronicity. Unhelpful illness perceptions (e.g., monocausal symptom explanation and expectation of long‐term symptom duration) and illness behaviours (e.g., ‘all or nothing’ and avoidance and control behaviours) have been shown to fuel perpetuation of such symptoms (Haines et al., 2019; Loades et al., 2019; Sullivan et al., 2019). Other suggested predictors of chronicity include symptoms from multiple organ systems, depressive symptoms, and parental functional somatic syndromes (FSS) (Janssens, Klis, et al., 2014). Without relevant treatment targeting modifiable perpetuating factors, adolescents risk far‐reaching long‐term consequences such as low educational attainment and increased risk of mental illness, and society may be burdened with high health care costs (Kashikar‐Zuck et al., 2014; Saunders et al., 2020).

The young patients may receive different diagnoses depending on main symptom presentation and attendance in health care. The most prevailing diagnoses include various FSS (e.g., juvenile fibromyalgia (JFM), chronic fatigue syndrome (CFS), functional gastrointestinal disorders (FGID), and idiopathic pain), somatoform disorders (ICD‐10) and somatic symptom disorder (SSD) (DSM‐5) (Garralda & Rask, 2015). However, despite an empirically proven overlap in symptomatology between these different diagnoses (Fink et al., 2007; Petersen et al., 2020a), the current classifications fail to cover the most severely affected patients with symptoms from multiple organ systems corresponding to multiple FSS (Burton et al., 2020).

Recent reviews show efficacy of psychological interventions, especially cognitive behavioural therapies (CBT), on various types of FSS in adolescents (Bonvanie et al., 2017; Fisher et al., 2018). Although psychological treatment has proven effective on specific FSS in adolescents, research regarding the most severely affected adolescents presenting with multiple FSS is scarce (Bonvanie et al., 2017). Studies on adults have shown efficacy of generic treatments of patients with multi‐organ symptom presentation (Fjorback et al., 2013; Schroder et al., 2012) and a recent feasibility study, though preliminary, also showed promising results in adolescents with multiple FSS (Ali et al., 2017).

Acceptance and Commitment Therapy (ACT) is a third wave intervention within the CBT tradition that has shown promising results in youth with chronic pain (Frostholm & Rask, 2019; Wicksell et al., 2009) and in adults with various FSS (Frostholm & Rask, 2019). ACT offers a behavioural health approach that differs from traditional treatments by facilitating resilience to symptoms and distress rather than focusing on symptom reduction. Herewith, ACT challenges inadequate coping strategies such as avoidance and control behaviour (e.g., school absenteeism and avoidance of physical activity) (Janssens, Oldehinkel, et al., 2014; Janssens et al., 2011). In ACT, an improvement in functioning is suggested to be facilitated by an increase of psychological flexibility, that is, the ability to act in accordance with values and long‐term goals also in the presence of unpleasant thoughts, feelings, and bodily sensations. As immediate symptom relief and long‐term symptom elimination may not be realistic in youth with multiple FSS, the clear focus on values may increase motivation for behavioural change (Plumb et al., 2009).

To accommodate the lack of treatment for adolescents with multiple FSS, we developed “Acceptance and Commitment Therapy for Health in Adolescents” (AHEAD). In an uncontrolled pilot study, we found this new treatment to be feasible with a clinically relevant improvement of physical health 3 months after end of treatment (Kallesøe et al., 2020).

The primary aim of this randomised trial was to evaluate the long‐term efficacy of AHEAD added to enhanced usual care (EUC) in improving physical health in adolescents with multiple FSS. EUC consisted of clinical assessment followed by a psychiatric consultation with general health promoting strategies. We hypothesised that by targeting unhelpful illness perceptions, illness behaviours, and psychological inflexibility, AHEAD + EUC would result in improved self‐reported physical health and reduced symptom severity as compared with the control condition (EUC) 12 months after randomisation (i.e., at 7‐month FU of AHEAD).

## METHOD

### Study design

This parallel‐group, single‐site randomised trial was carried out at a University Hospital Clinic in Denmark specialised in the management of FSS. The trial was approved by The Danish Data Protection Agency (no. 1‐16‐02‐290‐14) and the Committee of Health Research Ethics of Central Denmark Region (no. 1‐10‐72‐181‐14). Trial registration: ClinicalTrials.gov NCT02346071, was performed before commencement of the trial. A detailed description of the study protocol, assessment of patients and the AHEAD treatment program has been reported elsewhere (Kallesoe et al., 2016; Kallesøe et al., 2020).

### Participants

Patients between 15 and 19 years were referred from general practitioners, hospital departments or medical specialists for assessment and treatment of debilitating FSS. Referrals were screened for eligibility, and patients likely to meet study criteria were invited for assessment at the clinic.

To ensure inclusion of the most severely affected patients, the empirically based diagnostic category multi‐organ Bodily Distress Syndrome (BDS) (Fink & Schroder, 2010; Petersen et al., 2020b) was used as a diagnostic conceptualization of multiple FSS.

To be eligible for participation, individuals had to fulfill the diagnostic criteria for multi‐organ BDS (i.e. at least three functional somatic symptoms from at least three of four bodily systems) of at least 1 year's duration (Fink & Schroder, 2010) and clinician‐rated moderate to severe impairment based on distress, and impairment in daily life activities. Exclusion criteria were acute psychiatric disorder demanding other treatment, a lifetime diagnosis of psychosis, serious cognitive deficits or developmental disorders, substance abuse or pregnancy.

Oral and written consent was obtained from all participants before enrolment. Oral consent was obtained from parents.

### Randomisation and masking

Permuted block randomisation with seven blocks of 14–16 patients was performed. Patients were randomised to either AHEAD or EUC by means of a computer algorithm with seven to eight patients in each block allocated to AHEAD. Sealed opaque envelopes containing treatment allocation were prepared before enrolment of the first patient by an independent statistician. After assessment, eligible patients were enrolled. The assessment was performed by four physicians (specialists in child‐ and adolescent psychiatry (2), psychiatry (1), and social medicine (1), respectively), who also enrolled eligible patients. All four physicians performed EUC while the child‐ and adolescent psychiatrists also performed the AHEAD intervention. Masking of treatment allocation was not possible to the patients or physicians.

### Procedures

Potentially eligible patients underwent clinical assessment consisting of (1) Review of medical records and discharge letters, (2) Standardised clinical interview with history taking, (3) Semi‐structured diagnostic interview ‘Schedules for Clinical Assessment in Neuropsychiatry (SCAN)’ with a detailed section on functional somatic symptoms and screening for general psychopathology, (4) Screening for Attention Deficit Hyperactivity Disorder (ADHD), autism, and conduct disorder with sections from the Development and Well‐being Assessment (DAWBA) as child psychiatric disorders are not covered by SCAN, and (5) Clinical/neurological examination including routine blood tests (Kallesoe et al., 2016). Patients not eligible were given advice and recommended to contact their GP.

The physicians performing the assessment were certified in conducting the SCAN interview, and regular co‐ratings of video‐taped interviews were performed to ensure a high reliability in diagnostic procedures.

Data were collected through electronic questionnaires sent by e‐mail with no access for the project group before finalising 12‐month follow‐up of the last patient.

Questionnaires were distributed to patients at baseline (before assessment and randomisation) and at 2, 4, 5.5 (2 weeks after end of treatment (EOT)), 8 (3‐month follow‐up (FU)), and 12 months (7‐month FU). The distribution of questionnaires was designed to follow the group modules (i.e., before module 1, after module 8 and 9 and after follow‐up meeting). Parents received questionnaires at baseline and at 5.5 and 12 months aligned to their young person's assessment. A complete overview of questionnaires and time points of distribution is provided in the protocol (Kallesoe et al., 2016).

#### Enhanced usual care

Patients allocated to EUC received a 1½‐hour manualised psychiatric consultation with a physician providing further psychoeducation regarding multiple FSS and introducing general health promoting strategies for sleep, eating habits, physical and social activities, and engagement in positive activities. Recommendations for implementation including a treatment plan for the health care provider or general practitioner were issued.

An individual assessment of the need for subsequent involvement of psychologist, physiotherapist, school‐counsellor, or social services was made. Upon request, the physician would advise relevant professionals of strategies and support needed.

#### Acceptance and Commitment Therapy for Health in Adolescents (AHEAD)

Patients allocated to AHEAD received the above‐described psychiatric consultation prior to start of group therapy (i.e., AHEAD + EUC, from here on referred to as AHEAD). The manualised group‐based therapy was given in groups of seven to eight patients with nine modules (i.e., 27 h in total) over a period of 3 months and one follow‐up meeting (3 h) 3 months after the last module. Each module had an overall ACT‐related theme, for example “identification of personal values and barriers” and “avoiding the unpleasant and the long‐term consequences” to facilitate a behavioural change in accordance with values and long‐term goals (Kallesøe et al., 2020).

The treatment program was carried out by a total of 3 therapists, that is, two child‐ and adolescent psychiatrists and one psychologist, all with ACT training and specific knowledge on FSS. Each group had two therapists, that is, one of the child‐ and adolescent psychiatrists together with the psychologist.

### Outcomes

#### Primary outcome

##### Physical health

The primary outcome was an aggregate score measuring physical health derived from the SF‐36 subscales PF (physical functioning), BP (bodily pain) and VT (vitality) shown to be sensitive to change in key areas affected in adults with FSS (Bjorner et al., 1998; Rief et al., 2017; Schroder et al., 2012). Scores range from 15 to 65 with higher scores indicating better physical health. A change of 4 and above may be regarded as a clinically relevant change and 8 and above as a marked improvement (Ware et al., 1996; Ware & Kosinski, 2001). Gender and age‐specific Danish norm data are available from age 16 and up (Bjørner et al., 1997).

#### Secondary outcomes

*Overall impression of change* was measured with Patient Global Impression of Change (PGIC) (1 item, 7‐point scale) (Hurst & Bolton, 2004). Answers range from “no change (or condition has gotten worse)” to “a great deal better and a considerable improvement that has made all the difference”.

*Symptom severity* was measured by two questionnaires, that is, the somatization subscale of the Symptom Checklist Revised (12 items, 5‐point scale, and score range 0–4) (Derogatis & Cleary, 1977) and the BDS Checklist (25 items, 5‐point scale, and score range 0–100) (Budtz‐Lilly et al., 2015; Petersen et al., 2020) with higher scores indicating higher symptom load.

*Illness worry* was measured by Whiteley‐6 (6 items, 5‐point scale, and score range 0–4), a validated modified version of the Whiteley Index (Carstensen et al., 2020). Higher scores indicate more severe symptoms of illness worry.

*Emotional distress* was measured by SCL‐8, a subscale from Symptom Checklist Revised‐90 (8 items in total, 5‐point scale, and score range 0–4) (Christensen et al., 2010). Higher scores indicate a higher degree of emotional distress.

*Symptom impact* was evaluated with the limitation index (LI), a modified version of the Pain Interference Index (PII) (6 items, 7‐point scale, and score range 0–36) with higher scores indicating higher symptom impact (Holmstrom et al., 2015).

*Perceived stress* was assessed by the Perceived Stress Scale (PSS) (10 items, 5‐point scale, and score range 0–40) with higher scores indicating a higher degree of perceived stress (Cohen et al., 1983).

#### Treatment targets

*Illness perception* was measured by the Brief Illness Perceptions Questionnaire (BIPQ) (8 items, 11‐point scale, score range 0–80, and an open‐ended question regarding cause of symptoms) (Broadbent et al., 2015). A higher score reflects a more threatening view of the illness.

*Illness‐related behaviour* was measured by two subscales of the Behavioural Responses to Illness Questionnaire (BRIQ): (1) all‐or‐nothing behaviour (6 items, 5‐point scale, and score range 6–30) and (2) limiting behaviour (excessive rest) (7 items, 5‐point scale, and score range 7–35) with higher scores indicating a higher degree of maladaptive illness‐related behaviour (Spence et al., 2005).

*Psychological inflexibility* was measured by two questionnaires, that is, the brief version of Avoidance and Fusion Questionnaire in Youth (AFQ‐Y8) (8 items, 5‐point scale, and score range 0–32) (Greco et al., 2008) and the Psychological Inflexibility in Pain (PIPS‐12) (12 items, 7‐point scale, and score range 12–84) (Wicksell et al., 2010). In both questionnaires, higher scores indicate a higher degree of psychological inflexibility.

#### Expectations and satisfaction with treatment

After assessment and randomisation and prior to treatment start, patients completed questionnaires regarding experience of the assessment and their expectations of the upcoming treatment. At 5.5 months, the patients received questions regarding their satisfaction with the provided treatment.

#### Treatment adherence

The therapists' protocol adherence and treatment integrity were assessed in two ways that is, by independent observers and by two independent raters (see Supporting Information  S1).

#### Additional treatment

Information on additional treatment was obtained at the 12‐month visit, where the patients were asked if, and to which extent, they had received psychological treatment, physiotherapy, additional medication, and/or been in contact with social services.

#### Adverse events

Adverse events were self‐reported at 12 months. Clinicians continuously monitored group‐participants for potentially serious events.

### Statistical analysis

The sample size calculation was based on the aggregate score for physical health (SF36). Given results from two previous RCTs (Schroder et al., 2012; Wicksell et al., 2009), the improvement on physical health was estimated to 5 and 3 points in AHEAD + EUC and EUC respectively, given an SD of 8 at baseline. With a two‐sided alpha of 0.05 and a 95% power, we needed to allocate 60 patients to each group.

Descriptive statistics was used to characterize our sample at baseline. Depending on the distribution of the variables, data was summarised as either mean and standard deviation (SD), median and inter‐quartile range (IQR), or as count and percentage. The efficacy of the AHEAD intervention was analysed by means of a linear mixed models (LMM). That is, for the primary and each of the continuous secondary outcomes, we fitted a LMM with time, intervention group and their interaction as the primary explanatory variables. All mixed models included a random intercept. To account for the influence of the block randomisation, this was added as a random effect for the randomisation blocks. In all models, we first tested whether the two groups developed differently over time (i.e., an interaction effect of group and time), and next, as the main efficacy analysis pertains to the data at 12 months, we calculated the difference in change from baseline to 12 months between the two groups. All available data from all participants were included in the mixed models, and the models were checked by graphical inspection of the distribution of the residuals and random intercepts. Furthermore, for both groups, we computed the proportion of patients who had improved by at least 4 and 8 points on the SF‐36 aggregate score, between baseline and 8 and 12 months, respectively, and compared them by calculating the corresponding relative risks (RR). The PGIC was analysed with a proportional odds model with intervention group as the primary explanatory variable, and using this model, we calculated a risk difference (RD) comparing the probability of having scored ‘moderately better’, ‘better’ or ‘a great deal better’ in the two groups.

Both the LMM's and the proportional odds models were repeated while adjusting for the influence of age, gender, and parental education. The unadjusted results are found in Table S1. No imputation was considered necessary, due to low degree of missing data. All analyses were performed using Stata version 16.0 for Windows. The researchers and statistician were blinded to treatment allocation during performance of all unadjusted analyses. There was no data monitoring committee.

## RESULTS

### Trial profile and baseline characteristics

**FIGURE 1:**
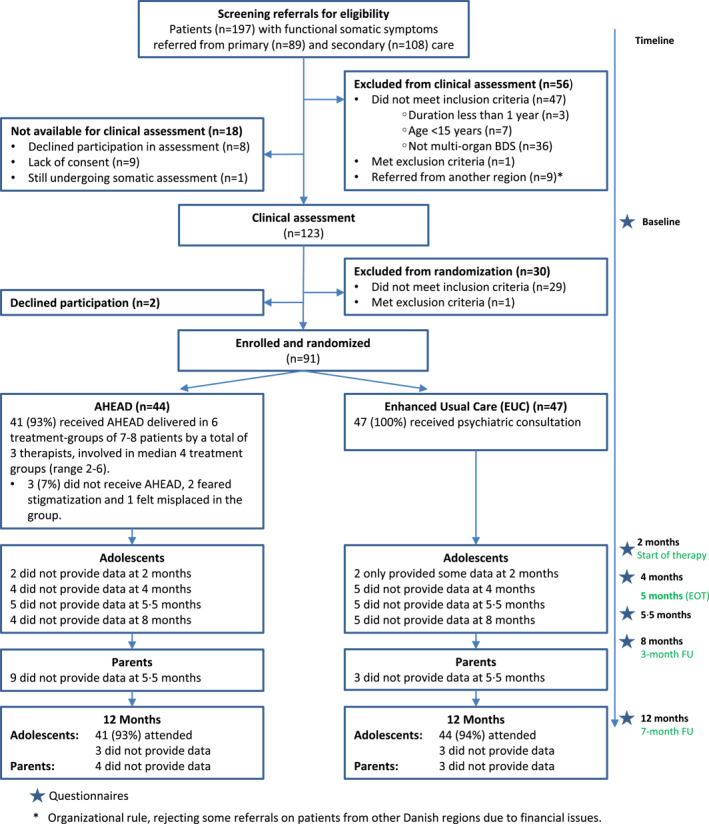
Trial profile

From 31 January 2015, to 10 December 2018, a total of 197 consecutively referred patients were screened for eligibility; 56 did not meet the criteria and 18 were not available for assessment (Figure 1). Thus, 123 participated in baseline clinical assessment. A total of 91 (98%) of 93 eligible patients were randomised: 47 to EUC and 44 to AHEAD. Ninety patients (99%) provided data at one or more time points after baseline, and 85 (93%) provided data at 12 months. Missing data were similar in both groups. One patient did not start the assigned treatment (AHEAD) due to being afraid of stigmatisation by receiving psychological treatment, and two patients dropped out of treatment after the second and fourth module, respectively. Inclusion was stopped before reaching the intended 120 patients due to a significantly slower recruitment rate than expected.

Table 1 shows baseline characteristics of both groups. Patients allocated to AHEAD and EUC were demographically and clinically similar, with a mean age of approximately 18 years. Ninety per cent of included patients were girls. Forty‐four per cent had psychiatric comorbidity such as anxiety, depression or attention deficit disorder, while 11% fulfilled the diagnosis for a dissociative disorder. Patients displayed a long mean symptom duration of approximately 4 years and had low self‐reported work‐capacity. A parental family history of FSS was reported by 35% and 44% reported a parental family history of psychiatric disorders.

**TABLE 1:** Patient characteristics

	AHEAD	EUC
Age at inclusion, years^a^	18.1 (1.5)	17.7 (1.5)
Sex, female, *n* (%)	40 (90.9%)	42 (89.4%)
Symptom duration, years^a^	4.3 (2.4)	3.5 (1.7)
Physical Health Aggregate Score^a^ ^,^ ^c^ (15–65)	36.6 (5.8)	37.1 (7.8)
Self‐rated labour capacity^a^ ^,^ ^d^ (0–10)	4.1 (0.3)	4.0 (0.4)
Symptom score SCL‐somatization^a^ (0–4)	2.0 (0.7)	1.8 (0.8)
Mental component score (MCS)^a^ ^,^ ^e^	35.7 (16.5)	35.8 (11.7)
Emotional distress^a^ ^,^ ^f^ (0–4)	1.7 (1.2)	1.9 (1.1)
Illness worry^a^ ^,^ ^g^ (0–4)	1.6 (1.1)	1.7 (1.1)
Limitation due to symptoms^a^ ^,^ ^h^ (0–36)	24.7 (7.0)	24.1 (8.0)
Primary ICD‐10 diagnosis, *n* (%):		
Somatization disorder (F 45.0)	23 (52.3%)	29 (61.7%)
Undifferentiated somatoform disorder (F 45.1)	21 (47.7%)	18 (38.3%)
Dissociative disorders (F 44.x), *n* (%):	5 (11.4%)	5 (10.6%)
Syndrome diagnoses fulfilled,^i^ *n* (%):		
Tension‐type headache	41 (93.2%)	45 (95.7%)
Non‐cardiac chest pain	36 (81.8%)	37 (78.7%)
Chronic fatigue syndrome (CFS)	35 (79.6%)	36 (76.6%)
Fibromyalgia	30 (68.2%)	29 (61.7%)
Irritable bowel syndrome (IBS)^j^	15 (34.1%)	16 (34.0%)
Psychiatric comorbidity total, *n* (%):	20 (45.5%)	20 (42.6%)
Anxiety disorders	15 (34.1%)	15 (31.9%)
Depressive disorders	12 (27.3%)	10 (21.3%)
Disorders of activity and attention	1 (2.3%)	2 (4.3%)
Clinician‐rated impairment, *n* (%):		
Moderate	11 (25.0%)	14 (29.8%)
Severe	33 (75.0%)	33 (70.2%)
School or work attendance, *n* (%):		
Normal conditions	1 (2.3%)	1 (2.1%)
High degree of absence, special conditions negotiated	37 (84.1%)	38 (80.9%)
No school or work attendance	6 (13.6%)	8 (17.0%)
Family status:		
Parents divorced, *n* (%)	15 (34.1%)	20 (42.6%)
Highest level of parental education *n* (%):		
<10 years	6 (13.6%)	8 (17.0%)
10–14 years	25 (56.8%)	31 (66.0%)
>14 years	12 (27.3%)	5 (10.6%)
Missing	1 (2.3%)	3 (6.4%)
Family history of^b^: *n* (%)		
Functional somatic syndrome (parents)	17 (38.6%)	15 (31.9%)
Functional somatic syndrome (extended family)	8 (18.2%)	9 (19.1%)
Psychiatric disorder (parents)	19 (44.2%)	21 (44.7%)
Substance abuse (parents)	5 (11.4%)	6 (13.3%)

^a^ Mean (SD).

^b^ Anamnestic information.

^c^ Aggregate score of the SF‐36 subscales: physical functioning, bodily pain, and vitality (range 15–65).

^d^ Self‐rated labour capacity (range 0–10, 10 being full capacity).

^e^ SF‐36 Mental Component Score.

^f^ SCL‐8.

^g^ Whiteley‐6.

^h^ Limitation index.

^i^ Post‐hoc analysis based on SCAN interview data.

^j^ Based on the Rome IV criteria. However, the SCAN interview does not include the item 'related to defecation', hence percentage with IBS is likely underestimated.

### Treatment expectations

Prior to treatment start, the patients rated their expectations of symptom improvement (scale 0‐100) after end of treatment. AHEAD patients had a mean expectation of 52.4 95% CI(44.4; 60.4), and EUC patients had a mean of 44.5 95% CI(35.7; 53.2), *p* = .379 (Figure S1) suggesting equipoise of treatment expectations.

### Psychiatric consultation and additional treatment

Patients in EUC received a higher degree of personally targeted intervention at the psychiatric consultation (Table S2) and significantly more treatment (i.e., psychological, physiotherapy, alternative treatment, social intervention, and/or pharmacological) outside the trial than patients in AHEAD as registered at 12 months (see Table S3). Especially additional psychological treatment differed (<0.001) between groups, with more than sixty percent of patients in EUC receiving psychological treatment with a median of 15 h, 95% CI (8; 25). Also, the use of over the counter pain medication and sleeping aids (sedatives and antipsychotics) differed between groups (*p* = .013 and *p* = .048, respectively) with patients in EUC receiving a higher degree of pharmacological treatment.

In the AHEAD intervention, the overall attendance rate of treatment was 91.6% (*n* = 41). Therapists spent an average of nine treatment hours per attending patient including group modules, individual consultation, and workshop for relatives.

### Primary outcome

**FIGURE 2:**
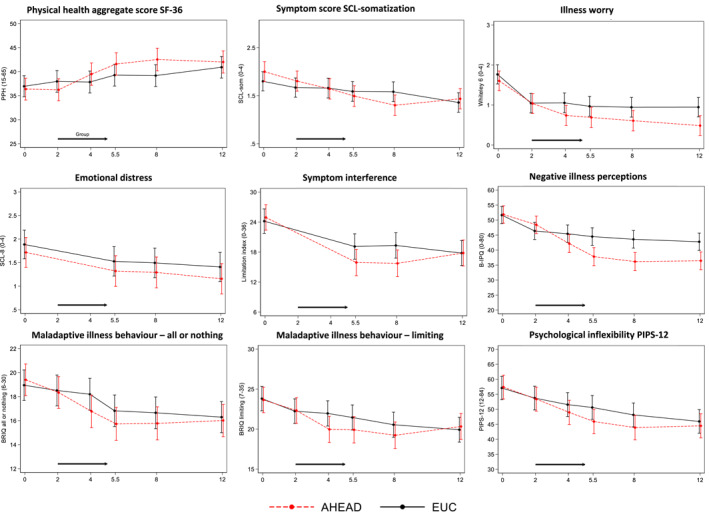
Adolescent reported outcomes. Physical health aggregate score (SF36) is primary outcome. Outcomes with only two measurement points (i.e., BDS checklist and PSS) or several measures for the same outcome (i.e., psychological inflexibility, AFQ‐Y8) are not presented in the figure but can be seen in Table S1. AHEAD, Acceptance and Commitment Therapy for Health in Adolescents; B‐IPQ, Brief Illness Perception Questionnaire; BRIQ, Behavioural Responses to Illness Questionnaire; EUC, Enhanced Usual Care; PPH, Perceived Physical Health SF‐36 aggregate score; PIPS‐12, Psychological Inflexibility in Pain Scale; SCL‐8, Symptom Checklist Rvised‐90—emotional distress subscale; SCL‐som, Symptom Checklist Revised‐90—somatization subscale

For the primary outcome analysis, a clinically relevant difference in improvement in physical health was seen for patients receiving AHEAD as compared to EUC (Δ = 3.3, 95% CI (0.5; 6.2), *p* = .023) at 8 months (Table S1). However, at the pre‐defined primary endpoint (12 months after randomisation corresponding to 7‐month FU), a relevant difference was no longer observed between interventions (Δ = 1.2, 95% CI(−1.6; 4.0), *p* = .404), primarily due to further improvement in the EUC group. Different developments over time were seen with an interaction effect between intervention arm and time (*χ* ^2^(5) = 14.1, *p* = .0148) (Figure 2). Within group improvements were 5.74 95% CI(3.69; 7.78) for AHEAD and 2.42 95% CI(0.44; 4.41) for EUC at 8 months and 5.22 95% CI(3.23; 7.21) for AHEAD and 4.03 95% CI(2.08; 5.99) for EUC at 12 months.

Table 2 and Figure S2 show percentages of patients experiencing clinically relevant treatment responses and decline on physical health at 8 and 12 months for both interventions with inclusion of between‐group comparisons (RR). At 12 months, 53.7% (95% CI (37.4; 69.3)) of patients in AHEAD displayed a relevant improvement and 36.6 95% CI (22.1; 53.1) a marked improvement while for patients in EUC, 50.0% 95% CI (34.6; 65.4) displayed a relevant improvement and 31.8% 95% CI (18.6; 47.6) a marked improvement.

**TABLE 2:** Change in physical health (SF‐36 aggregate score) from baseline to 8 and 12 months

	AHEAD		EUC		Comparison 8 months	Comparison 12 months
	8 months	12 months	8 months	12 months	AHEAD versus EUC	AHEAD versus EUC
	(*n* = 38)	(*n* = 41)	(*n* = 42)	(*n* = 44)	RR 95% CI *p* value	RR 95% CI *p* value
Marked decline^a^	2.6	2.4	4.8	11.4	0.55 (0.05; 5.85) .622	0.21 (0.03; 1.76) .152
(≤−8 physical health) % (95% CI)	(0.1; 13.8)	(0.1; 12.9)	(0.6; 16.2)	(3.8; 24.6)		
Decline	7.9	14.6	21.4	13.6	0.37 (0.11; 1.26) .112	1.07 (0.38; 3.06) .895
(≤−4 physical health) % (95% CI)	(1.7; 21.4)	(5.6; 29.2)	(10.3; 36.8)	(5.2; 27.4)		
Treatment response	55.3	53.7	38.1	50.0	1.45 (0.89; 2.34) .129	1.07 (0.71; 1.62) .736
(≥4 physical health) % (95% CI)	(38.3; 71.4)	(37.4; 69.3)	(23.6; 54.4)	(34.6; 65.4)		
Marked improvement^b^	34.2	36.6	11.9	31.8	2.87 (1.13; 7.31) .027	1.15 (0.64; 2.08) .643
(≥8 physical health) % (95% CI)	(19.6; 51.4)	(22.1; 53.1)	(4.0; 25.6)	(18.6; 47.6)		

*Note*: The proportion of patients that has not experienced neither decline nor treatment response (i.e., change in physical health >4 and <4) is not specifically reported.

Abbreviations: AHEAD, Acceptance and Commitment Therapy for Health in Adolescents; CI, confidence interval; EUC, enhanced usual care; RR, relative risk.

^a^ Subset of the group of patients reporting a decline, to evaluate the proportion with marked decline.

^b^ Subset of the group of patients reporting a treatment response, to evaluate the proportion with marked improvement.

### Secondary outcomes and treatment targets

Secondary outcome analyses are reported in Figure 2 and Table S1. Patient Global Impression of Change (PGIC) showed a risk difference for having a score indicating a clinically relevant improvement of Δ  = 27.7%, 95% CI(10.5; 44.8), *p* = .002 at 5.5 months, Δ  = 26.3%, 95% CI(8.0; 44.5), *p* = .005 at 8 months and Δ  = 25.3%, 95% CI (6.9; 43.7), *p* = .007 at 12 months in favour of AHEAD. Analysis of PGIC as reported by parents showed a risk difference of Δ  = 42.2%, 95% CI (24.7; 59.6), *p* ≤ .001 at 5.5 months and Δ  = 29.9%, 95% CI (12.5; 47.3), *p* = .001 at 12 months in favour of AHEAD. Unadjusted answers are presented in the Figure S3.

At 12 months, an improvement was seen in both groups on all other secondary outcomes, with no clinically important between‐group differences in change from baseline to 12 months. For SCL‐somatization and Limitation Index, a difference in developments over time was seen in favour of AHEAD with interaction effects between intervention arm and time (i.e., SCL‐somatization (*p* = .006) and limitation index (*p* = .039)). Furthermore, a difference in developments over time was seen on Limitation Index as reported by parents in favour of AHEAD with interaction effect between intervention arm and time (*p* = .026).

**FIGURE 3:**
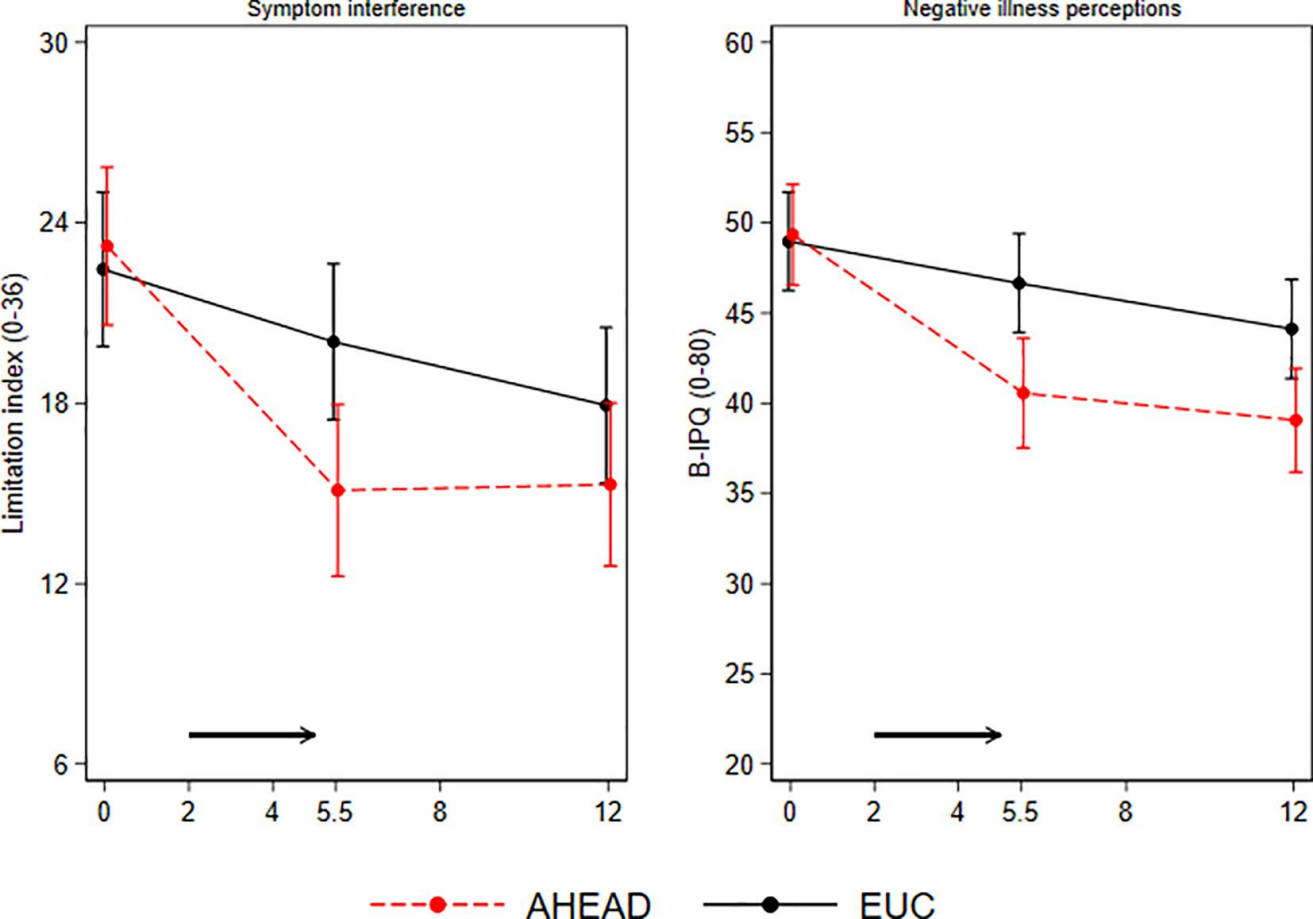
Parental reported outcomes. Symptom interference and negative illness perceptions from parental report

AHEAD patients displayed a larger decline in the treatment target 'maladaptive illness perceptions' with a between‐group difference in change of −5.9 95% CI (−10.0; −1.8), *p* = .005. No clinically important between‐group difference was observed on illness behaviour or psychological inflexibility. From parent reports, a between‐group difference was seen on maladaptive illness perception at 12 months with a larger decline in AHEAD (*p* = .018) (Figure 3).

### Treatment satisfaction and adverse events

Treatment satisfaction was rated at 5.5 months (2 weeks after end of treatment) where 34 (87.2%) patients in AHEAD and 12 (28.6%) patients in EUC rated their treatment offer as good or excellent. Ratings of bad or unacceptable were 1 (2.6%) in AHEAD and 18 (42.9%) in EUC, *p* < .001 (Figure S4).

Five patients in AHEAD and 5 in EUC reported experiencing adverse events. No serious adverse events were reported. In AHEAD, two commented that getting more in contact with feelings elevated their levels of anxiety. In EUC, one described feeling excessively medicalised and two reported stress due to not receiving any help.

## DISCUSSION

According to recent reviews, psychological treatment has been found effective for youth with specific FSS, but there is a lack of empirically supported treatments for patients with multi‐organ symptom presentation (Bonvanie et al., 2017; Fisher et al., 2018). Data from this randomised trial showed that AHEAD, a generic group‐based therapy for adolescents with co‐occurrence of multiple FSS, performed equally well as EUC 12 months after randomisation (i.e., seven months after treatment was finished). Notably, AHEAD patients had a faster improvement in physical health and symptom severity, and impact compared to EUC. Also, EUC patients reported receiving more psychological treatment outside the trial indicating that most EUC patients experienced a need for additional treatment. Furthermore, both patients and parents expressed overall improvement more often after AHEAD than after EUC and showed important changes in illness perception (a predefined treatment target) at most measurement points. Additional gains of providing AHEAD were less use of pharmacological treatment and less use of other non‐documented treatment forms such as alternative treatment where some of it potentially could be harmful for the patients.

As both interventions resulted in significant long‐term effects, results from this clinical trial underpins the importance of a systematic clinical approach for young patients with multi‐organ symptom presentations, and that an accurate diagnosis, evidence based psychoeducation and early intervention is an important first step towards improvement. Furthermore, the high treatment satisfaction in AHEAD suggests that specialized psychological treatment is helpful and meaningful for these patients.

Results from this study are in line with findings from two previous randomised trials of internet‐ and family‐delivered CBT for youth with CFS respectively, where long‐term follow‐up did not show superiority of intervention in regards to primary outcome (Chalder et al., 2010; Lloyd et al., 2012; Nijhof et al., 2013). In one of the trials using psychoeducation as the control condition, results showed a faster return to school in the intervention group (Chalder et al., 2010) and also maintained improvements in emotional and behavioural difficulties in the intervention group at follow‐up, as opposed to a deterioration in the control group (Lloyd et al., 2012). The faster improvement of the AHEAD patients seen in this trial may have similar implications on for example, school absence, which should be explored in future studies.

The results from the present study indicate that a generic approach is feasible for youth with multiple FSS. Current evidence for psychological treatment of youth is predominately limited to treatment of specific types of FSS (Bonvanie et al., 2017; Fisher et al., 2018) lacking uniformity in terms of diagnostic categories including patients with multi‐organ symptom presentation and generic treatment offers across symptomatology as proposed in recent adult literature (Burton et al., 2020; Henningsen et al., 2018; Schröder et al., 2015). This implies a risk of current health care organisations providing fragmented and insufficient care for youth, and that more severely affected patients with multi‐organ symptom presentation are neglected (Kangas et al., 2020).

The results from the present study needs to be interpreted in the light of some limitations.

First, masking of patients and therapists regarding treatment allocation was not feasible, imposing a risk of placebo effect in AHEAD and nocebo effect in EUC. However, before entering the two treatment arms, the patients' treatment expectations with regard to symptom reduction were almost the same in AHEAD and EUC suggesting equipoise between the two groups at treatment start.

Second, the trial included fewer than the planned 120 patients due to a specified timeframe of the study and a slower recruitment than expected, which may have resulted in the analyses being underpowered to detect significant differences between the groups.

Third, there was a potential contamination due to having the same physicians delivering both AHEAD and EUC treatment, with a risk of inclusion of ACT elements in the EUC arm. Although the EUC treatment was manualised the psychiatric consultation (EUC) was not video‐taped, which implies a need to evaluate this in more detail in the future.

Strengths of the present study supporting the validity of the results include a randomised design with a relatively large sample compared to other studies in youth, taking symptom profile with multi‐organ presentation and symptom duration into account (Bonvanie et al., 2017; Fisher et al., 2018). All patients participated in a thorough assessment by a physician using validated diagnostic measures. Comorbid anxiety and depression was allowed which increases generalisability of the findings, as emotional comorbidities are common in the patient group (Garralda & Rask, 2015). The treatment was manualised, and adherence to the manual and ACT as a therapeutic concept was systematically evaluated by independent raters from a random sample of all video‐taped sessions.

Of importance for further development, some patients receiving AHEAD experienced a decline in physical health at follow‐up and the EUC group caught up with the AHEAD group at 12 months. This implies a need to extend the support beyond 3 months in order to stabilise achieved improvements. Also, it may be important to identify patients who are in need of even more comprehensive treatment. This argument is supported by a number of other studies in the field where some young patients report long‐term continuation of symptoms despite relevant treatment (Rowe, 2019; van der Veek et al., 2013). Whether a next step in line for the patients with long‐lasting disorder is extended follow‐up to maintain helpful strategies, a larger degree of involvement of family, school and social services as seen with other chronic patient groups in youth (Howard et al., 2016), and/or more interdisciplinary and intensive treatment for those most severely affected (Heimann et al., 2018) currently remains unclear. However, early diagnosis, ongoing support including help to stay engaged in the school system (Rowe, 2020) and timely intervention seem pivotal to increase the potential of recovery (Nijhof et al., 2013).

In summary, the AHEAD intervention resulted in a clinically relevant improvement that remained at 12 months' follow‐up, for adolescents with multiple FSS. This improvement was similar to the EUC control group at 12 months, that is, no significant interaction effect between time and group were found. However, AHEAD patients showed a faster improvement on physical health and secondary outcomes including symptom severity, symptom impact, and negative illness perception. In addition, patients in EUC received considerably more psychological and pharmacological treatment outside the trial. The results indicate that a generic intervention with inclusion of young patients with different dominant symptoms and symptom profiles may be feasible. Overall, this may be an important step forward in a field where many patients are reluctant to accept psychological treatment for FSS, and where treatment offers for youth are scarce and diverse.

## CONFLICT OF INTEREST

The authors have declared that they have no competing or potential conflicts of interest.

## ETHICS STATEMENT

The trial was approved by The Danish Data Protection Agency (no. 1‐16‐02‐290‐14) and the Committee of Health Research Ethics of Central Denmark Region (no. 1‐10‐72‐181‐14).

## AUTHOR CONTRIBUTIONS

Conceptualization of the project was done by Karen H. Kallesøe, Andreas Schröder, and Charlotte Ulrikka Rask. The methodology was decided by Karen H. Kallesøe, Andreas Schröder, Rikard K. Wicksell, and Charlotte Ulrikka Rask. Formal analysis and investigation were done by Karen Hansen Kallesøe, Jens Søndergaard Jensen, and Charlotte Ulrikka Rask. Karen H. Kallesøe prepared the original draft and Andreas Schröder, Rikard K. Wicksell, Jens Søndergaard Jensen, and Charlotte Ulrikka Rask reviewed and edited the draft. Funding was acquired by Karen Hansen Kallesøe and Charlotte Ulrikka Rask. Supervision on the trial was done by Andreas Schröder, Rikard K. Wicksell and Charlotte Ulrikka Rask. Role of the funding source: The funders of the study (i.e., TrygFonden (grant number 100408) and The Danish Medical Association (grant number 2013‐5480/912523‐63)) had no role in study design, data collection, data analysis, data interpretation, or writing of the report. All authors had full access to the full data in the study and had final responsibility for the decision to submit for publication.

## Supporting information

Supporting Information S1

## Data Availability

The individual participant data that underlie the results reported in this article will be available for researchers who provide a methodologically sound proposal to achieve their aims in the approved proposal. Proposals should be directed to corresponding author karkal@rm.dk; to gain access. Data requestors will need to sign a data access agreement. The study protocol, statistical analysis plan and analytic code are available. The data are available immediately following publication and no specific end date has been set.
