# Supplementary material for: Group‐based Acceptance and Commitment Therapy (AHEAD) for adolescents with multiple functional somatic syndromes: A randomised trial
Source: JCPP Adv. 2021 Dec 8;1(4):e12047. doi: 10.1002/jcv2.12047 (PMC10242822; doi:10.1002/jcv2.12047)
Supplement: Supplementary file 1 — Supporting Information S1 [file JCV2-1-e12047-s001.docx]

**Supporting Information for Kallesøe et al.**

**Figure S1. Treatment expectations**

**
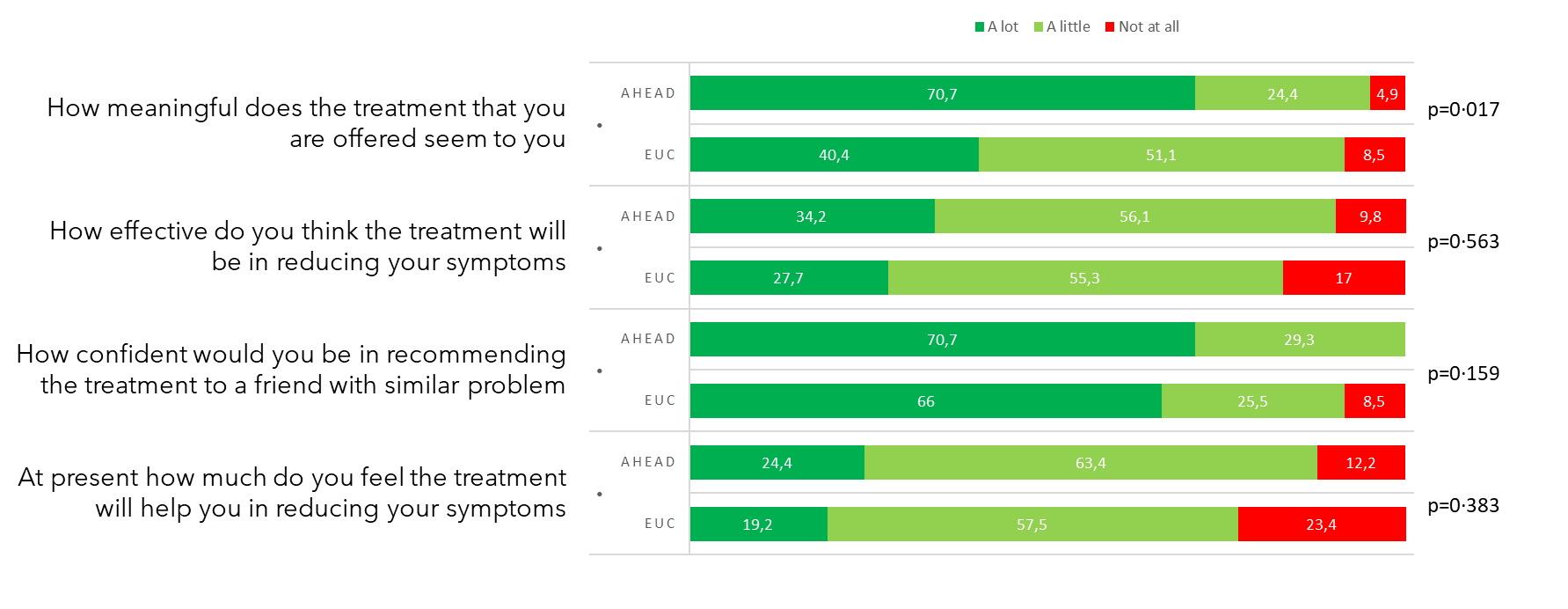
**

Treatment expectations as reported by the patients after assessment but before attending the psychiatric consultation (i.e. EUC)

**Figure S2. Change in physical health from baseline to 8 and 12 months**


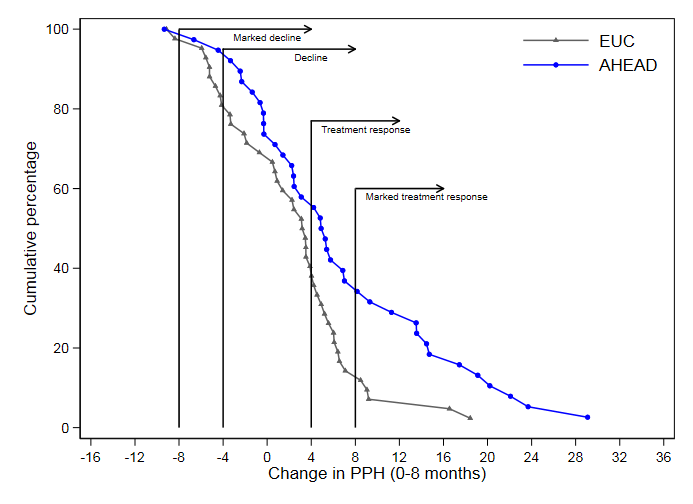

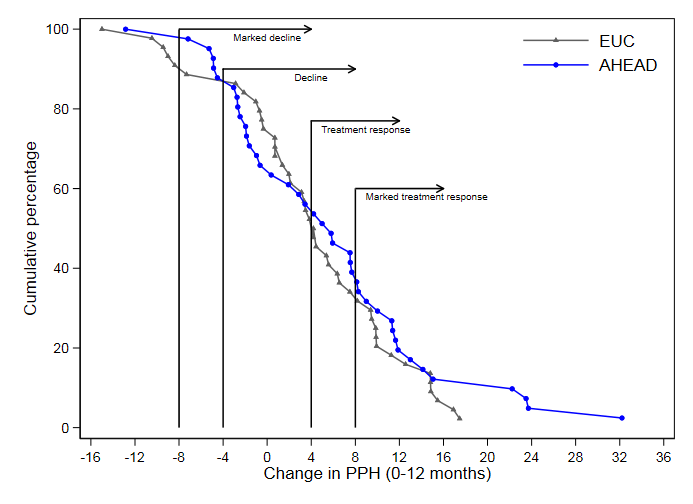


Plots present the observed data, with each dot representing the observed change score for an individual patient who provided data at 8 and 12 months. Vertical lines indicate the levels of improvement: treatment response change in physical health ≥4, marked treatment response change in physical health ≥8, decline change in physical health ≤-4, marked decline ≤-8.

**Figure S3. Patient Global Impression of Change (PGIC)**

Global impression of change (unadjusted) as reported by patients and parents

**Figure S4. Treatment satisfaction at end of treatment (5.5 months)**

**Table S1. Improvement and difference on primary and secondary outcomes**

Adj., Adjusted; AHEAD, Acceptance and Commitment Therapy for Health in Adolescents; AFQ-Y8, Avoidance and Fusion Questionnaire for youth; B-IPQ, BDS, Bodily Distress Syndrome; Brief Illness Perception Questionnaire; BRIQ, Behavioural Responses to Illness Questionnaire; CI, Confidence Interval; Diff., Difference; EUC, Enhanced Usual Care; PPH, Perceived Physical Health SF-36 aggregate score; PIPS-12, Psychological Inflexibility in Pain Scale; PSS, Perceived Stress Scale; SCL-8, SF-36, Short Form Health Survey; Symptom Checklist Rvised-90 – emotional distress subscale; SCL-som, Symptom Checklist Revised-90 – somatisation subscale; Unadj., Unadjusted.

**Table S2. Provided treatment in relation to the EUC consultation**

| Recommendation on: | AHEAD | EUC | p-value* |
| --- | --- | --- | --- |
| Medication  (e.g. reduction in pain medication) | 2  (4.6%) | 6  (12.8%) | 0.166 |
| Psychological treatment  (advice to seek treatment outside the trial) | 0 | 21  (44.7%) | <0.001 |
| Physiotherapy  (advice to seek treatment outside the trial) | 3  (6.8%) | 13  (27.7%) | 0.009 |
| **Contact to**:** |  |  |  |
| School by physician  (e.g. advice on reduction of hours or need for extra support) | 20  (45.5%) | 21  (44.7%) | 0.941 |
| Social services by physician  (e.g. recommendations to social services of need for individual or family based interventions) | 0 | 18  (38.3%) | <0.001 |

*p-value for difference is tested with x^2^

** Written or by telephone

The table presents five areas addressed at the psychiatric consultation (i.e. medication and need of additional help from psychologist, physiotherapist, the school or social services).

**Table S3. Additional treatment at 12 months**

| Treatment | AHEAD | EUC | p-value* |
| --- | --- | --- | --- |
| Psychological n (%),  median [IQR] | 8 (19.0%);  4 hrs [3;8] | 27 (61.4%);  15 hrs. [8;25] | 0.001  <0.001 |
| Physiotherapy n (%),  median [IQR] | 9 (22.0%);  6 hrs. [5;10] | 21 (47.7%);  4 hrs. [2;12] | 0.049  0.033 |
| Alternative treatment*  (e.g. acupuncture, cranio sacral therapy) n (%) | 11 (26.2%) | 21 (47.7%) | 0.048 |
| Intervention from social services n (%) | 3 (7.1%) | 7 (15.9%) | 0.219 |
| Pharmacological treatment: |  |  |  |
| Pain medication, over the counter (e.g. paracetamol, ibuprofen) | 2 (5.0%) | 11 (25.0%) | 0.013 |
| Pain medication, prescription (e.g. opioids or cannabidiol) | 0 (0.0%) | 3 (6.8%) | 0.088 |
| Relief of abdominal symptoms (e.g. antacid, emetics and laxatives) | 3 (7.5%) | 8 (18.2%) | 0.159 |
| Antidepressants | 7 (17.5%) | 7 (15.9%) | 0.809 |
| Sleeping aid  (e.g. sedatives, antipsychotics) | 0 (0.0%) | 4 (9.1%) | 0.048 |

*p-value for difference is tested with chi^2^ for percentages and Wilcoxons ranksum test for hours

AHEAD, Acceptance and Commitment Therapy for Health in Adolescents; EUC, Enhanced Usual Care; IQR, Interquartile range

**Appendix**

**Assessment of protocol adherence and therapist competences**

The therapists' protocol adherence and treatment integrity were assessed in two ways. Firstly, two independent observers (1 psychologist and 1 child- and adolescent psychiatrist) attended two different groups with different setups of therapists. From a manual-developed checklist of all treatment elements (86 elements), they registered each specific element being performed. Furthermore, treatment integrity (ACT consistency (5 items) and inconsistency (4 items), overall adherence to treatment manual (1 item) and therapist competency (1 item) for ACT were assessed using a scoring guideline specifically developed for clinical studies on ACT (score range 1-5) (Plumb & Vilardaga, 2010). Higher scores indicate higher ACT consistency, higher ACT inconsistency, and higher competence, respectively. All treatment modules, i.e. 1-9, in all groups were video-taped. A total of 9 group meetings (17%) ensuring representation of all modules and representation of the different groups (1-6) were randomly selected for evaluation. Two independent ACT-trained psychologists performed the ratings.

**Results**

Adherence to 86 predefined treatment elements (6-13 elements at each module) from the manual was rated by two independent observers in group 3 and group 6, respectively. In both groups, a total of 84 elements (97.7%) were addressed in treatment. In the evaluation of treatment integrity from video-taped sessions, the ACT-consistent items received a mean rating of 4.80 (SD 0.19) and the ACT-inconsistent items received a mean rating of 1.14 (SD 0.18). Overall competence of therapists received a mean rating of 4.89 (SD 0.32). For further details, see Table S4.

**Table S4. Treatment integrity**

| Rater 1 | ACT consistency | |  |  |  | ACT inconsistency | |  |  | Overall adherence | Competence of therapist |
| --- | --- | --- | --- | --- | --- | --- | --- | --- | --- | --- | --- |
| Session | **Item 1** | **Item 2** | **Item 3** | **Item 4** | **Item 5** | **Item 6** | **Item 7** | **Item 8** | **Item 9** | **Item 10** | **Item 11** |
| 1 | 5 | 5 | 5 | 4 | 4 | 1 | 1 | 1 | 1 | 4 | 5 |
| 2 | 5 | 5 | 5 | 5 | 3 | 1 | 1 | 1 | 1 | 5 | 5 |
| 3 | 4 | 5 | 5 | 5 | 5 | 1 | 2 | 1 | 2 | 4 | 4 |
| 4 | 5 | 5 | 5 | 5 | 5 | 1 | 2 | 1 | 1 | 4 | 5 |
| 5 | 5 | 5 | 5 | 5 | 5 | 1 | 2 | 1 | 1 | 5 | 4 |
| 6 | 5 | 5 | 4 | 5 | 5 | 1 | 2 | 1 | 1 | 4 | 5 |
| 7 | 5 | 5 | 3 | 4 | 5 | 1 | 1 | 1 | 1 | 5 | 5 |
| 8 | 5 | 5 | 5 | 5 | 5 | 1 | 1 | 1 | 1 | 5 | 5 |
| 9 | 5 | 5 | 4 | 5 | 5 | 1 | 1 | 1 | 1 | 5 | 5 |

Nine sessions were rated as seen in the table. Item 1-5 ACT consistency, item 6-9 ACT inconsistency, item 10 overall adherence to project manual and item 11.

| Rater 2 | ACT consistency | |  |  |  | ACT inconsistency | |  |  | Overall adherence | Competence of therapist |
| --- | --- | --- | --- | --- | --- | --- | --- | --- | --- | --- | --- |
| Session | **Item 1** | **Item 2** | **Item 3** | **Item 4** | **Item 5** | **Item 6** | **Item 7** | **Item 8** | **Item 9** | **Item 10** | **Item 11** |
| 1 | 5 | 5 | 4 | 5 | 4 | 1 | 1 | 1 | 2 | 5 | 5 |
| 2 | 4 | 5 | 5 | 5 | 4 | 1 | 1 | 1 | 1 | 5 | 5 |
| 3 | 4 | 5 | 5 | 5 | 5 | 1 | 1 | 1 | 1 | 5 | 5 |
| 4 | 5 | 5 | 5 | 5 | 5 | 1 | 1 | 1 | 1 | 4 | 5 |
| 5 | 5 | 5 | 5 | 5 | 5 | 1 | 2 | 1 | 1 | 5 | 5 |
| 6 | 5 | 5 | 4 | 5 | 5 | 1 | 2 | 1 | 1 | 5 | 5 |
| 7 | 5 | 5 | 3 | 5 | 5 | 1 | 1 | 1 | 1 | 5 | 5 |
| 8 | 5 | 5 | 5 | 5 | 5 | 1 | 2 | 1 | 2 | 5 | 5 |
| 9 | 5 | 5 | 5 | 5 | 5 | 1 | 1 | 1 | 1 | 5 | 5 |

Nine sessions were rated as seen in the table. Item 1-5 ACT consistency, item 6-9 ACT inconsistency, item 10 overall adherence to project manual and item 11.

Treatment integrity was evaluated using a scoring protocol developed for ACT studies (Plumb & Vilardaga, 2010) with minor adjustments as used in a previous trial in FSS (Kemani et al., 2015). Two licensed psychologists with ACT training performed the ratings. The raters received training in the scoring protocol and a joint test-rating of a module from the pilot study was conducted together with KHK before rating the selected modules.

The items in the treatment scoring included ACT consistency (item 1-5) and inconsistency (item 6-9), overall adherence to project manual was (item 10) and competence of therapist (item 11). Score range on all items was 1-5.

Due to the small number of sessions rated and very low inter-rater variation the planned interrater reliability analysis was not feasible. Instead raw data are provided.

Kemani, M. K., Olsson, G. L., Lekander, M., Hesser, H., Andersson, E., & Wicksell, R. K. (2015). Efficacy and Cost-effectiveness of Acceptance and Commitment Therapy and Applied Relaxation for Longstanding Pain: A Randomized Controlled Trial. *Clin J Pain*, *31*(11), 1004-1016. <https://doi.org/10.1097/ajp.0000000000000203>

Plumb, J. C., & Vilardaga, R. (2010). Assessing treatment integrity in acceptance and commitment therapy: Strategies and suggestions. *International Journal of Behavioral Consultation and Therapy*, *6*(3), 263-295. <https://doi.org/10.1037/h0100912>
